# Effects of habitat fragmentation on the population genetic diversity of the Amur minnow (*Phoxinus lagowskii)*

**DOI:** 10.1080/23802359.2017.1331319

**Published:** 2017-06-07

**Authors:** Zhuang Xue, Yu-Ying Zhang, Mao-Shang Lin, Shi-Meng Sun, Wei-Feng Gao, Wei Wang

**Affiliations:** a College of Fisheries and Life Science, Dalian Ocean University, Dalian, China;; b Department of Biological Sciences, Florida International University, North Miami, FL, USA

**Keywords:** Habitat fragmentation, reproduction isolation, Amur minnow, DNA (D-loop), genetic diversity

## Abstract

*Phoxinus lagowskii* is a freshwater fish that is widely distributed in China. In this study, a comparative analysis of the mtDNA control region (D-loop) was performed to analyze the natural population structure and genetic diversity of 54 individuals from eleven locations (T1, T2, T3, T4, T5, T6, T7, T8, T9, T10 and T11) which was divided with reservoirs. The estimated haplotype and nucleotide diversity were 0.734 and 0.03514, respectively. An AMOVA indicated that 79.78% of the total variation originated from individual populations and 20.22% came from variation within the 11 geographic populations, which showed high genetic differentiation among the 11 geographic groups. A test of neutral evolution and mismatch distribution indicated that historical expansion occurred in these populations. However, the findings of low genetic diversity and high genetic differentiation demonstrated that the reproduction isolated by reservoir has showed a certain effect for the development of the populations, and the results should provide new information for the conservation and exploitation of this species.

## Introduction

The Amur minnow (*Phoxinus lagowskii*), a member of the family Cyprinidae, subfamily Leuciscus, is a common edible fish that is abundant and widely distributed in the Yangtze River, Qingjiang River, Hanjiang River and northern Yellow River in China (Guo & Wu 2012; Wang et al. 2013). Additionally, as the dominant species, the Amur minnow is widely distributed in the Liaohe River, the sampling site of the present study. A segment of the migratory route of the Amur minnow was sealed off by a glacier during the last glaciations, isolating a subpopulation of this species; thus, the Amur minnow is not contiguously distributed in streams, leading to its long-term isolation in the reservoir (Wang et al. 2005; Ye et al. 2005). Thus, *Phoxinus lagowskii* is an ideal model organism for the study of the geographic formation of the Liaohe River.

In recent years, due to a variety of human-caused threats and environmental pollution, the number of Amur minnow has decreased rapidly (Zhang 2006). Because of their very limited numbers and their high sensitivity to environmental factors, in 1988, this species was listed as a second-class state-protected wild animal in the China Red Data Book of Endangered Animals (Yue & Chen 1998). However, information on this species remains very scarce, and it is urgent for researchers to develop methods to protect this prized fish resource as soon as possible (Zhao & Zhang 2009; Kang, et al. 2011; Si et al. 2012). And with the completion of several reservoirs in the Liaohe River before 70s of the last century (Dong & Shi 1989), production isolation should be take place on the populations of *Phoxinus lagowskii*. How serious is the risk for the genetic diversity of *Phoxinus lagowskii*? The study of the population dynamics of *Phoxinus lagowskii* is important for the conservation of its endemic germplasm resources and genetic diversity.

As an effective molecular marker due to its rapid rate of evolution and maternal inheritance (Sun et al. 2012), mitochondrial DNA (mtDNA) has been widely applied to the study of population genetic structure, the origin and evolution of species, and genetic relationships(Weiss et al. 2011; Shi et al. 2012; Liu et al. 2015). In the present study, the mitochondrial control region (D-loop) was used to analyze the population genetic diversity and adaptive variation of the *Phoxinus lagowskii* from 11 populations in Liaoning Province. The results will be useful for the management of the germplasm resource, biodiversity conservation and exploitation and for better understanding the effects of habitat fragmentation on the genetic structure of the species to lay the foundation for effective protection and management strategies.

## Materials and methods

### Materials

**Figure 1.**
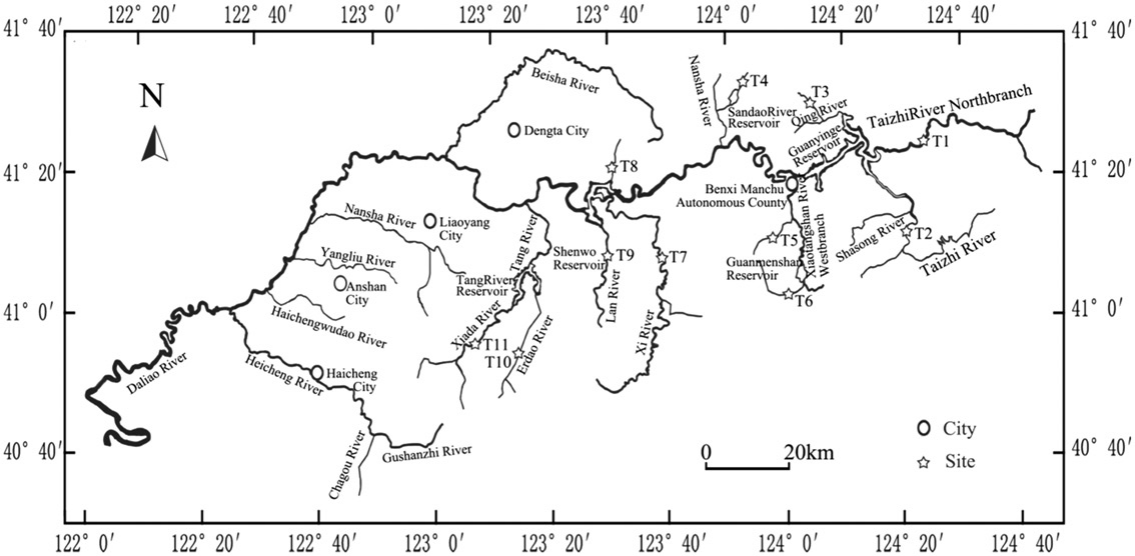
Sampling locations of the 11 *Phoxinus lagowskii* populations.

Adult *Phoxinus lagowskii* were obtained in December from 11 different sampling points that at least were separated by a reservoir in the Liaohe River (Figure 1), Liaoning Province, China. Fin clips were collected and stored in 95% ethanol until DNA extraction.

### Extraction of genomic DNA

Total genomic DNA was extracted from the fin tissue samples by a modified phenol/chloroform extraction method (Sambrook et al. 1989): the ethanol-preserved fin tissues (0.1 g) were dried for 30 min at 70 °C and then washed twice with ddH_2_O, followed by digestion in 600 ml of tissue extract (50 mM Tris–HCl (pH 8.0), 100 mM ethylenediaminetetraacetic acid (EDTA) (pH 8.0), 1% SDS) and 5–8 ml 20 mg/ml proteinase K for 4 h at 50 °C with constant gentle mixing. After centrifugation at 12,000 rpm for 10 min, the supernatant (DNA extract) was collected in a new 1.5-ml microcentrifuge tube and was sequentially extracted with 300 ml each of phenol and chloroform. The total DNA was recovered by overnight precipitation in 1 ml volumes of frozen absolute ethanol and washed twice by 75% ethanol at 12,000 rpm. The total DNA was standardized to a concentration of 50 ng/ml and stored in ddH_2_O.

### Amplification of D-loop sequences

The mitochondrial D-loop was amplified by polymerase chain reaction (PCR) using the forward primer DL1: 5'-caa cat gcc ggg cgt tca tg-3' and the reverse primer DL2: 5'-gcg atg gct aac cgt agc tc-3'. Amplifications were carried out in 30 μl reaction volumes containing the following components: 4 μl of genomic DNA, 23.2 μl of golden mix (Golden Easy PCR System; TaKaRa, Dalian, China), 1.2 μl of each primer, 0.4 μl of Taq polymerase (TaKaRa, Dalian, China). The PCR conditions were as follows: 95 °C for 3 min followed by 35 cycles of denaturing at 94 °C for 30 s, 55 °C for 30 s and 72 °C for 1 min, and a final extension at 72 °C for 10 min. All of the amplified products were purified with a Gel Extraction Kit (Generay Biotech (Shanghai) Co., Ltd., Shanghai, China) following the manufacturer’s instructions. The PCR products were sent to Shanghai Generay Biotech Co. Ltd. (Shanghai, China) for sequencing.

### Sequence alignments and analyses

The sequences were proofread, edited and aligned with BioEdit version 7.0.5.3 (Ibis Biosciences, Carlsbad, CA). The polymorphism was assessed using DnaSP version 5.0 (Universitat de Barcelona, Barcelona, Spain). Arlequin software version 3.11 (University of Berne, Berne, Switzerland) was used to estimate the genetic differentiation and Fst values with an analysis of molecular variance (AMOVA) model (Excoffier et al. 2005). The historical population expansion was examined using Tajima’s (1989) and Fu’s (Fu 1997) statistics with 10,000 demographic history records among the four populations. The parameters of the genetic diversity within and between populations, such as the haplotype diversity (Hd), nucleotide diversity (Pi), average number of nucleotide difference (K) and gene flow (Nm), were estimated with MEGA software (version 5.05), and the phylogenetic reconstruction of the haplotypes was also conducted using the neighbour-joining (NJ) method performed using MEGA version 5.05 from a bootstrap consensus of 10,000 replicates (Kumar et al. 2004; Tammura et al. 2007).

## Results

### Composition analysis of the D-loop sequence

Approximately 300 bp of the mtDNA control region of 11 populations was obtained (Figure 1), which shared high homology with other cyprinid fishes. A total of 54 sequences were analyzed, and the estimated transition/transversion bias (R) was 5.00, A = 29.6, C = 27.8, G = 14.9 and T = 27.8, with (A + T) (57.4%) significantly higher than (C + G) (42.6%) (Table 1).

**Table 1.** Base composition of the mitochondrial D-loop gene sequence of eleven populations of *Phoxinus lagowskii*.

Population	T	C	A	G	T + A	G + C
T1	28.3	27.7	29.3	14.7	57.6	42.4
T2	28.1	27.8	29.5	14.7	57.6	42.5
T3	28.2	27.8	29.3	14.7	57.5	42.5
T4	28.2	27.5	29.6	14.7	57.8	42.2
T5	28.0	27.9	29.4	14.6	57.4	42.5
T6	27.5	26.7	30.9	15.0	58.4	41.7
T7	26.6	28.1	29.9	15.4	56.5	43.5
T8	27.0	28.6	28.2	16.2	55.2	44.8
T9	27.9	27.1	30.3	14.6	58.2	41.7
T10	28.1	27.9	29.1	14.8	57.2	42.7
T11	28.0	27.9	29.4	14.7	57.4	42.6
Mean value	27.8	27.7	29.6	14.9	57.4	42.6

### Analysis of genetic diversity and population genetic structure

Twenty-five D-loop haplotypes from 54 individuals (*Phoxinus lagowskii*) (Table 2) were obtained using DnaSP software version 5.0. Haplotype Hap_2 was shared among nine populations, Haplotype Hap_1 was separately observed in the T2 and T11 populations, and the other haplotypes were unique to each population. Five haplotypes were observed in the T7 population, which is the most in any of the populations, and only one haplotype was observed in the T3 population, which is the least.

**Table 2.** Distribution of the 25 haplotypes in *Phoxinus lagowskii* populations.

Haplotype	T 1	T 2	T 3	T 4	T5	T6	T7	T8	T9	T10	T11
Hap_1	–	1	–	–	–	–	–	–	–	–	1
Hap_2	4	4	3	3	2	3	–	–	1	4	4
Hap_3	–	–	–	1	–	–	–	–	–	–	–
Hap_4	–	–	–	–	1	–	–	–	–	–	–
Hap_5	–	–	–	–	–	1	–	–	–	–	–
Hap_6	–	–	–	–	–	–	1	–	–	–	–
Hap_7	–	–	–	–	–	–	1	–	–	–	–
Hap_8	–	–	–	–	–	–	–	1	–	–	–
Hap_9	–	–	–	–	–	–	–	1	–	–	–
Hap_10	–	–	–	–	–	–	–	1	–	–	–
Hap_11	–	–	–	–	–	–	–	1	–	–	–
Hap_12	–	–	–	–	–	–	–	–	–	1	–
Hap_13	–	–	–	–	–	–	1	–	–	–	–
Hap_14	–	–	–	–	–	–	1	–	–	–	–
Hap_15	–	–	–	–	1	–	–	–	–	–	–
Hap_16	–	–	–	1	–	–	–	–	–	–	–
Hap_17	–	–	–	–	–	–	–	–	–	1	–
Hap_18	–	–	–	–	–	–	–	–	–	1	–
Hap_19	–	–	–	–	–	–	–	–	1	–	–
Hap_20	–	–	–	–	–	–	–	–		–	1
Hap_21	–	–	–	–	–	–	–	–	1	–	–
Hap_22	–	–	–	–	–	1	–	–	–	–	–
Hap_23	–	–	–	1	–	–	–	–	–	–	–
Hap_24	–	–	–	–	–	–	1	–	–	–	–
Hap_25	–	–	–	–	–	1	–	–	–	–	–

The estimates of haplotype diversity and nucleotide diversity are shown in Table 3. The T7, T8 and T9 populations showed the highest haplotype diversity (*h* = 1.000), followed by the T5 population (*h* = 0.833), the T4 and T6 populations (*h* = 0.8), the T10 and T2 populations (*h* = 0.714; *h* = 0.7, respectively), with the T3 population exhibiting the lowest haplotype diversity. The total haplotype diversity of the T11 population was 0.734. The T7 population exhibited the highest level of nucleotide diversity (*π* = 0.08142) among the 11 populations.

**Table 3.** Haplotype diversity (*h*) and nucleotide diversity (π) of *Phoxinus lagowskii* populations.

Population	Haplotype diversity (*h*)	Nucleotide diversity (π)
T1	0.400	0.00155
T2	0.700	0.00388
T3	0.000	0.00000
T4	0.800	0.04131
T5	0.833	0.00388
T6	0.800	0.06219
T7	1.000	0.08142
T8	1.000	0.05184
T9	1.000	0.05168
T10	0.714	0.01350
T11	0.600	0.00259

### Analysis of molecular variance

The AMOVA model (Meirmans 2006) was used to test the significance of the 11 population structures based on the haplotype frequency; 20.22% of the molecular variance was attributed to the differentiation among populations, whereas 79.78% of the molecular variance was observed within populations (Table 4).

**Table 4.** Analysis of molecular variance (AMOVA) for the *Phoxinus lagowskii* populations.

Markers	Source of variation	Percentage of total variation	*P*
D-loop	Population	20.22	<.001
	Within population	79.78	<.001

**Figure 2.**
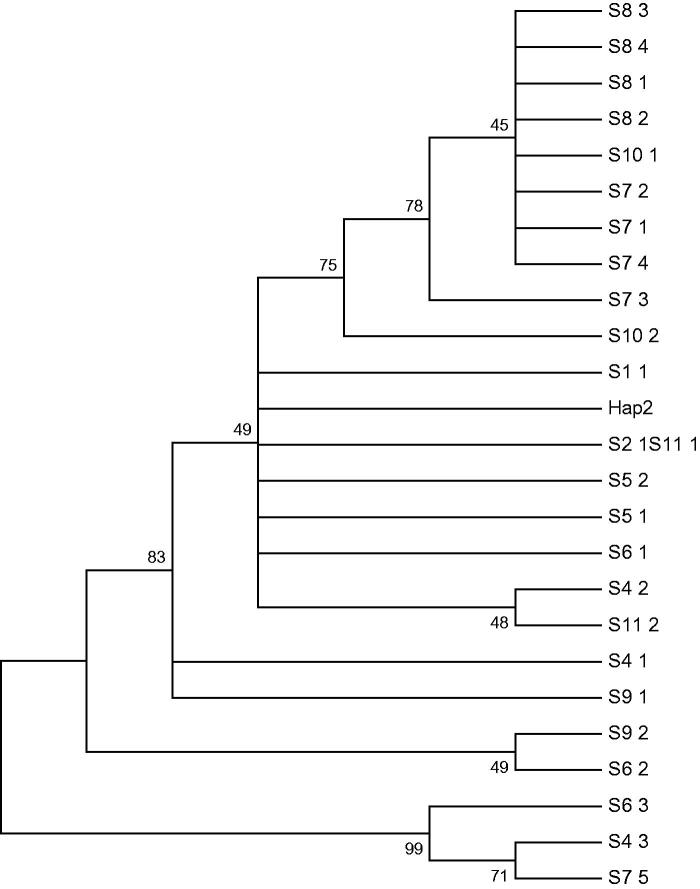
Neighbour-joining tree of mtDNA D-loop haplotypes of *Phoxinus lagowskii*. Numbers at the nodes are bootstrap values above 40%.

The tests of neutral evolution (Tajima’s D and Fu’s Fs) indicated a positive value (in population T8), but there was no significant difference (*p* > .05) among the 11 populations; the topology of the resulting neighbour-joining (NJ) tree (Figure 2) revealed that population T8 is relatively closed, and the other populations are dispersed. The above analysis indicated no significant population expansion in the 11 populations.

## Discussion

### Analysis of the structure of the mtDNA control regions of *Phoxinus lagowskii*

*Phoxinus lagowskii* is mainly distributed in the Heilongjiang River, Tumen River, Liaohe River, Yellow River and the middle reaches of the Yangtze River in China. *Phoxinus lagowskii* is the dominant carp species in the Liaohe River of Liaoning Province (Zheng & Zhang 2002).

The MtDNA control region sequences of 54 individuals were investigated and analyzed in this study. The D-loop region sequence is located between the tRNA-Pro and tRNA-Phe genes. The conserved sequence domain structure of the control regions showed high similarity with that reported for other Cyprinidae, i.e. *Phoxinus oxycephalus, Rhynchocypris lagowskii, Rhynchocypris oxycephalus* and *Phoxinus steindachneri* (Wang et al. 2011) The nucleotide microsatellite has also been found in some mammals and teleosteans (Yue et al. 2006); for example, Cyprinid fish possessed an AT-type microsatellite in the mtDNA D-loop. The differences in the nucleotide microsatellite can be used for the identification of separate families of fishes.

### Analysis of the genetic diversity of *Phoxinus lagowskii*

Twenty-five haplotypes were obtained based on the 54 individuals. The major haplotype (Hap_2), as a common haplotype among the nine populations, supports the fact that these populations are descended from a common ancestor, whereas the other twenty-four haplotypes separately belong to different populations. All twenty-five haplotypes were associated with the dispersion and expansion of the population (Figure 1). Therefore, the eleven populations appear to have diverged from each other in recent evolution. The cluster analysis supported the view that the fish were able to pass through the reservoir in the artificial drainage due to their smaller body size and subsequently mixed with populations from other regions, enabling gene flow, which explains the haplotype cross-phenomenon. We could infer this from the situations of the 11 populations that the population has experienced growth, reproduction and population growth in recent years. The result is consistent with the hypothesis that the subspecies has undergone a long isolation and evolutionary bottleneck (Miracle & Campton 1995). The results of the evolutionary analysis not only exhibited some intraspecific differences but also showed divergent and adaptive variance resulting from the long isolation of the different geographic populations and suggested that eight populations have experienced a long isolation and genetic divergence compared to other populations.

The analysis of the genetic structure of the eleven populations revealed that haplotype and nucleotide diversities were an important index of population genetic diversity. The haplotype and nucleotide diversities of the 54 individuals collected from the eleven sampling points were 0.7340 and 0.03514, respectively. The T7, T8 and T9 populations exhibited the highest haplotype diversity (*h* = 1.000), whereas the T7 population exhibited the highest nucleotide diversity (*π =* 0.08142). The *Phoxinus lagowskii* in the Liaoning River exhibit high genetic diversity. Nevertheless, this rare fish resource should be protected from the degeneration of biodiversity.

In recent years, populations of *Phoxinus lagowskii* have suffered severe population declines; in the past 40 years, some populations have totally disappeared due to increases in the local water temperature and excessive fishing by humans (Liang 2009).The distribution range of this species is shrinking. National and local natural reserves (Liaohe River Natural Reserve in Panjin) have been established by local governments to protect the ecosystems and biodiversity. The population of the *Phoxinus lagowskii* in the Liaohe River has adapted to changing environmental and demographic events (Sun et al. 2016). In recent years, many water drawdown projects have been implemented on the Liaohe River; although these projects have had no impact on fish populations in the short term, they will lead to the reduction of biodiversity of endangered species and even extinction in the long term. Therefore, it is important to consider gene exchange when water conservancy projects are constructed to ensure the sustainable development of the genetic resources of the biological population.

The differentiation of haplotypes in *Phoxinus lagowskii* conformed with the results of the AMOVA revealing that variation within the population accounted for 79.78% of the total variation and variation among populations was only 20.22%; therefore, genetic variation does not occur primarily in individual populations. Thus, it was concluded that the genetic differentiation among the eleven populations was low. Despite the low level of genetic differentiation, the results of the present study also support substantial differences in geographic structure associated with different population drainages. As above, the results of this research demonstrated that the reproduction isolated by reservoir has showed a certain effect for the development of *Phoxinus lagowskii* populations, and the results should provide some new information to the government and scientific researchers for the conservation and exploitation of this species.
